# The polymorphism and geographical distribution of knockdown resistance of adult *Anopheles sinensis* populations in eastern China

**DOI:** 10.1186/s12936-019-2793-z

**Published:** 2019-05-07

**Authors:** Wei-Long Tan, Chun-Xiao Li, Rui-Chen Lv, Yan-De Dong, Xiao-Xia Guo, Dan Xing, Ming-hao Zhou, Yan Xu, Hong-liang Chu, Gang Wang, Chang-qiang Zhu, Jun Sun, Tong-Yan Zhao

**Affiliations:** 1 State Key Laboratory of Pathogen and Biosecurity, Beijing Institute of Microbiology and Epidemiology, Beijing, 100071 China; 2 Department of Vector Control, Huadong Research Institute for Medicine and Biotechnics, Nanjing, 210002 Jiangsu China; 3 Department of Vector Control, Jiangsu Center for Disease Prevention and Control, Nanjing, Jiangsu China

**Keywords:** *Anopheles sinensis*, Knockdown resistance, *kdr* mutation, Pyrethroids

## Abstract

**Background:**

*Anopheles sinensis* is one of the major malaria vectors in China and other southeast Asian countries, including Vietnam, Cambodia, Thailand. Vector control is considered to be the critical measure for malaria control, while the increasing prevalence of insecticide resistance caused by long-term use of insecticides, especially pyrethroids, is threatening the successful control of *An. sinensis*. In order to understand the underlying resistance mechanisms involved and molecular basis, the principal malaria vector, *An. sinensis* from Jiangsu and Anhui provinces, Southeast China, was investigated.

**Methods:**

The adult *Anopheles* mosquitoes were sampled from multiple sites across Jiangsu and Anhui provinces, and sufficient mosquitoes collected from eleven sites for insecticide susceptibility bioassays. The DIIS4–DIIS6 region of the *para*-type sodium channel gene was amplified and sequenced, then multiple PCR and Taqman assays were used to assess the frequencies of *kdr* mutations at the target gene.

**Results:**

In the present study, most of the adult *An. sinensis* populations were pyrethroids resistant, which indicated the presence of *kdr* resistance mutations in the *para*-type sodium channel gene. Sequence analyses demonstrated the *kdr* mutation existed at codon 1014 in Jiangsu and Anhui provinces. In adult *An. sinensis*, three mutant types (TTT L1014F, TTC L1014F, and TGT L1014C) of *kdr* alleles were detected, while no wild type (TTG L1014) was observed. The TTC L1014F mutation was first reported in Anhui province.

**Conclusions:**

The highly polymorphic *kdr* alleles were observed in all the adult *An. sinensis* populations, which suggested that in-depth studies are required for carrying on insecticide resistance monitoring and specific resistance mechanisms studying into establish effective long-term malaria vector control program in eastern China.

## Background

Malaria is considered to be one of the most deadly vector-borne disease, which causes millions of malaria cases worldwide every year [1]. Despite huge efforts conceded for its elimination, about 3.2 billion people living in more than 100 countries worldwide are still at risk, especially children [2]. The mosquito *Anopheles sinensis* is considered to be the main malaria vector in mainland China [3, 4] and other Southeast Asian countries [5]. As no effective vaccine or anti-malarial drugs are available for malaria, the key strategy for disease prevention relies on the control of mosquito populations or reduction in human-vector contact. In the past few decades, the pyrethroid pesticides were widely used as a conventional control strategy for both larval and adult mosquito control for their low toxicity to humans, high efficacy against mosquito vectors and short residual action [6]. Recommend by the World Health Organization (WHO), the main strategies for adult mosquito control are indoor residual spraying (IRS) and insecticide-treated mosquito nets (ITNs). During the malaria outbreaks, these strategies were the most effective vector control measures to reduce the density of adult mosquitoes [7]. Selection pressure caused by long-term intensive use of insecticides is the key driving force in resistance development. Additional parameters such as environmental conditions may affect both the mosquito response to insecticides and the selection of resistance mechanisms.

Pyrethroid products are the mainly insecticides recommended by the WHO for current malaria control programmes. However, *Anopheles* resistance to pyrethroids and other kinds of insecticides is rapidly expanding in many malaria vectors including *Anopheles gambiae* and *Anopheles funestus* in Africa, *Anopheles minimus*, *Anopheles sinensis*, and *Anopheles dirus* in Asia, and *Anopheles darlingi* in South America. Furthermore, vector control programmes are always threatened by the development of insecticide resistance [8, 9]. Resistance to multiple pyrethroids insecticides has been reported in southern and eastern China in the larval stage [10–12], in Vietnam and Laos, North Korea, The Republic of Korea and Japan in the adult stage. Data on the distribution and evolution of pesticide resistance in adults of this species is urgently required in order to implement effective and sustainable control of this important disease vector.

DDT (dichloro-diphenyl-trichloroethane) and pyrethroids insecticides act by disrupting the voltage-gated sodium channel (VGSC) of the insect nervous system and making non-synonymous mutation(s) in VGSC gene to reduce the sensitivity, which has been referred as knockdown resistance (*kdr*) mutation [13]. Knockdown resistance mutations, providing resistance to DDT and pyrethroid insecticides in several insect species, have been linked to a single amino acid residue substitution in the *para*-type sodium channel structural protein [14] with resistance phenotype. Molecular characterizations have revealed that several mutations in the S1–S6 transmembrane fragments of domain II of the sodium channel gene confer resistance against DDT and pyrethroid insecticides in a number of insect species [15, 16], such as *Culex pipiens pallens* [17] and *An. gambiae* [18]. In *An. sinensis*, two point mutations of the voltage-gated sodium channel gene (L1014F and L1014C) have been found in southeast China and populations of Korea [19–21]. Both two mutations are associated with DDT and pyrethroid resistance in *An. sinensis* larvae [19]. In previous studies, co-existing of *kdr* mutations (TTG/TTT and TTG/TCG) in the VGSC gene which caused L1014F and L1014S substitutions has been reported in *An. sinensis* in the Guiping area, Guangxi province and Vietnam, Cambodia and Laos [13, 22]. Although it is acknowledged that insecticide resistance may differ between the aquatic and adult life stages, and that resistance in larvae is not always transferred to adults and vice versa, larval bioassays are still generally used for resistance monitoring in China. Because there is currently no information on the evolution and characteristics of *kdr* in adult *An. sinensis*, the WHO PES-approved procedure (Wire-Ball assay) was used to examine the susceptibility to commonly used insecticides and the distribution of *kdr* mutations in adult *An. sinensis* collected from Jiangsu and Anhui provinces [20]. Resistance phenotype is thought to be the important index for mosquito control with insecticides, and it is necessary to improve an exact genotyping assay to ease *kdr* frequency monitoring for giving insecticide resistance level.

## Methods

### Study sites

**Fig. 1:**
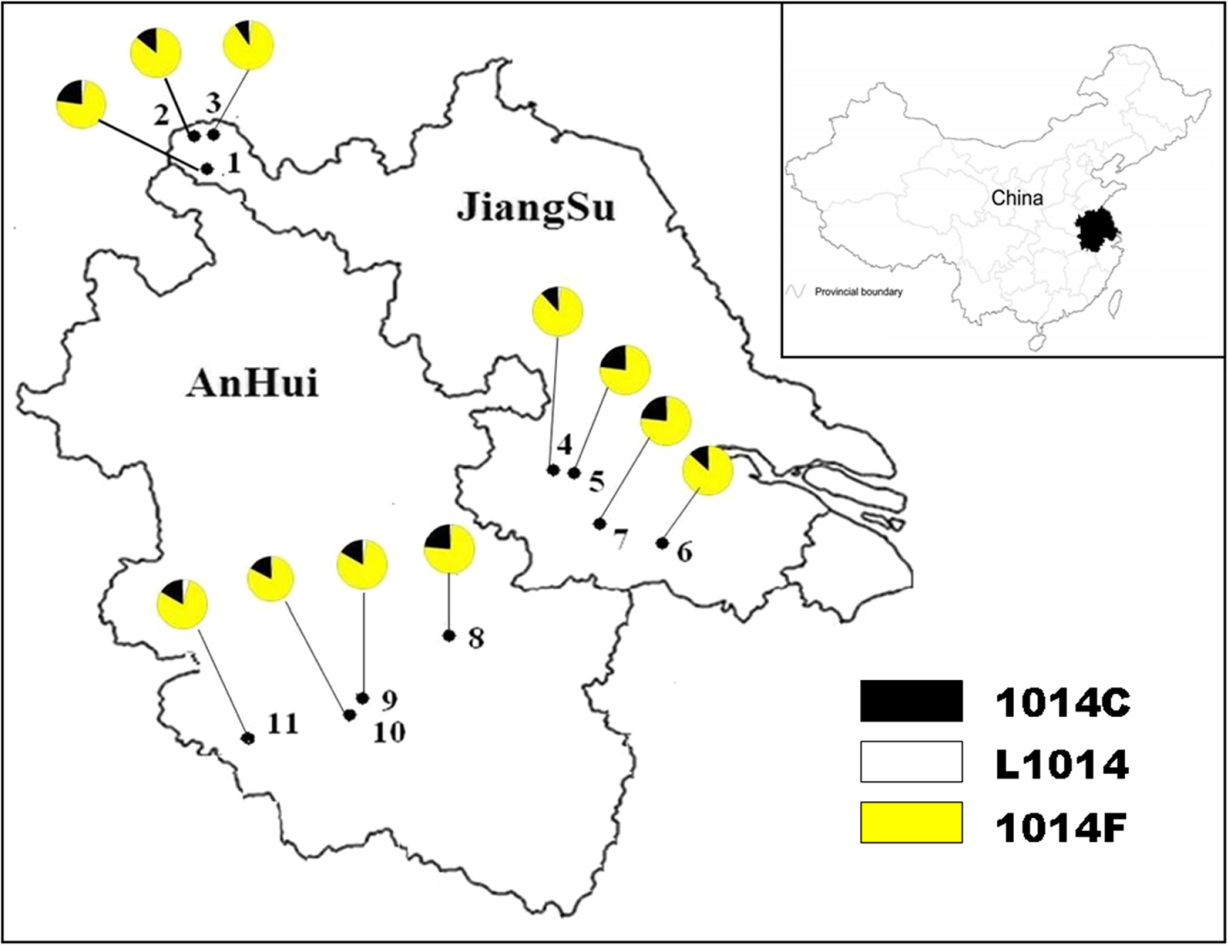
The location of collection sites of adult *Anopheles sinensis* specimens and distribution of *kdr* allele. 1: YL (Yunlong); 2: XN (Xiaonian); 3: SZ (Shazhuang); 4: DT (Dangtu); 5: DY (Danyang); 6: BN (Benniu); 7: CS (Changshu); 8: FC (Fanchang); 9: CZ (Chizhou); 10: WH (Wanghe); 11: PZ (Panzheng). In the colour circles, different colour indicated different substitutions in percentage

Specimens of adult *An. sinensis* were collected from eleven different geographical sites from 2009 to 2012, seven in Jiangsu province and four in Anhui province. The eleven sites will hereafter be referred to as YL(Yunlong), XN (Xiaonian), SZ (Shazhuang), DT (Dangtu), DY (Danyang), BN (Benniu), CS (Changshu), FC (Fanchang), CZ (Chizhou), WH (Wanghe) and PZ (Panzheng) (Fig. 1, Table 1).

**Table 1:** Sites where *An. sinensis* specimens were collected in Anhui and Jiangsu provinces, China

Collection site	Code	Collection method	Sample size	Latitude/longitude coordinates	Ecology	Date
Xuzhou, Yunlong (YL)	YL	IR-HC	66	34°79′041″N, 117°18′374″E	Paddy field	7/10
Fengxian, xiaonian (XN)	XN	IR-HC	72	34°54′949″N, 116°43′199″E	Paddy field	7/10
Fengxian, shazhuang (SZ)	SZ	IR-HC	48	34°54′941″N, 116°37′568″E	bullpen	7/10
Zhengjiang, Dangtu (DT)	DT	IR-HC	72	32°09′520″N, 119°32′803″E	Paddy field	7/10
Zhengjiang, Danyang (DY)	DY	IR-HC and IR-PSC	26	32°03′631″N, 119°50′903″E	Paddy field	7/10
Changzhou, Changshu (CS)	CS	IR-HC and LC	49	31°24′630″N, 119°47′900″E	Paddy field	7/10
Changzhou, Benniu (BN)	BN	IR-HC	72	31°52′076″N, 119°49′506″E	Paddy field	8/10
Fanchang, e-shan (FC)	FC	IR-HC	72	31°02′010″N, 118°15′040″E	Paddy field	8/10
Chizhou, Meilong (CZ)	CZ	IR-NET	72	30°77′130″N, 117°67′640″E	Pigsty	8/10
Qianshan, Wanghe (WH)	WH	IR-NET	68	30°48′810″N, 117°45′660″E	Pigsty	8/10
Qianshan, Panzheng (PZ)	PZ	IR-NET	72	30°42′010″N, 116°33′340″E	Pigsty	8/10

IR-HC, hand-operated aspirators, indoor collection; IR-NET, indoor resting mosquitoes in bed-nets; IR-PSC, pyrethrum spray collection; LC, larval collection

### Mosquito collection

All mosquitoes were adults collected in rural areas (primarily cowsheds, paddy fields and pigsties) and transported to the Insectaria of the Huadong Research Institute for Medicine and Biotechnics on the day of collecting, 293 Zhongshan East Road, Nanjing, Jiangsu province, China. After bioassays were completed (the F1 filial generation were also used in trials in some sites because of insufficient adult mosquitoes were collected.), all samples were stored at − 20 °C for molecular assay. A susceptible strain had been reared under laboratory conditions and protected from contact with insecticides for 20 years served as the control in pesticide resistance assays.

### Bioassay

A Wire-Ball bioassay was used to test the half knockdown time (KT_50_) of adult mosquito samples from the eleven sample sites according to WHO PES-approved procedures. This is a simple apparatus comprised of part of an intact mosquito net with an effective insecticide concentration of 100 mg/m^2^ wrapped around a frame comprised of two intersecting circles of wire of about 15 cm in diameter. A sleeve is made in the netting so that mosquitoes can be introduced and removed with an aspirator. The experience using this method suggests that the median knockdown time of mosquitoes exposed to impregnated nets may be faster than the 3 min reported by the WHO (CTD/WHOPES/IC/96.1). This method allowed adult mosquitoes to be tested in three parallel groups so only 33 adult female mosquitoes were needed for tests. Where insufficient adult mosquitoes were collected from some site specimens of the F1 filial generation were used in trials. A piece of netting without insecticide was used as a control; the survival rate of mosquitoes in which was greater than 95%.

### Mosquito DNA extraction

DNA was isolated from individual mosquitoes using the method developed by Livak and a TaKaRa DNA isolation kit (TaKaRa Agarose Gel DNA Purification Kit Ver.2.0, Code DV805A). Prior to DNA isolation, one-third of the lower-abdomen of female mosquitoes was removed and stored in liquid nitrogen in order to avoid DNA contamination from sperm in the reproductive tract.

### Genotyping the *kdr* gene by sequencing

The II S4–II S6 fragment of the *para*-type sodium channel gene was targeted to identify candidate sites for *kdr* mutations; the traditional *kdr* substitution loci, L1014F, L1014C [19] and L1014S [22]. For the *kdr* alleles, L1014, L1014F and L1014S is the the most common mutation in *An. gambiae* [23] and *Culex pipiens pallens* [17] and L1014F and L1014H is common in *Drosophila* [24]. The position of amino acids was numbered according to the partial sodium channel sequence documented for *An. sinensis* (GenBank accession number: JN002364.1).

*Kdr* allele genotypes of individual specimens were confirmed by DNA sequencing. The sequencing results showed that there was no alternative splicing in the sodium channel cDNA sequence and intervening sequence of *An. sinensis.* Fragments, including the *kdr*-F and *kdr-*C mutations regions of the *para*-type sodium channel gene from 110 wild templates (10 samples from each sampling site) and 10 control specimen templates were amplified using P1 and P2 primers based on the partial voltage-gated sodium channel (VGSC) genomic DNA sequence of *An. sinensis* (access number: DQ334052.1). Fragments of the genomic DNA of 10 wild specimens from each collection site and 10 control specimens were amplified with a Universal Genomic DNA Extraction Kit Ver.3.0 (Code DV811A Lot CA1401 Exp.Dec.2011, TaKaRa) and direct sequenced to identify sodium channel gene mutations.

### Multiple-PCR assay for genotyping *kdr* allele and species identification

**Fig. 2:**
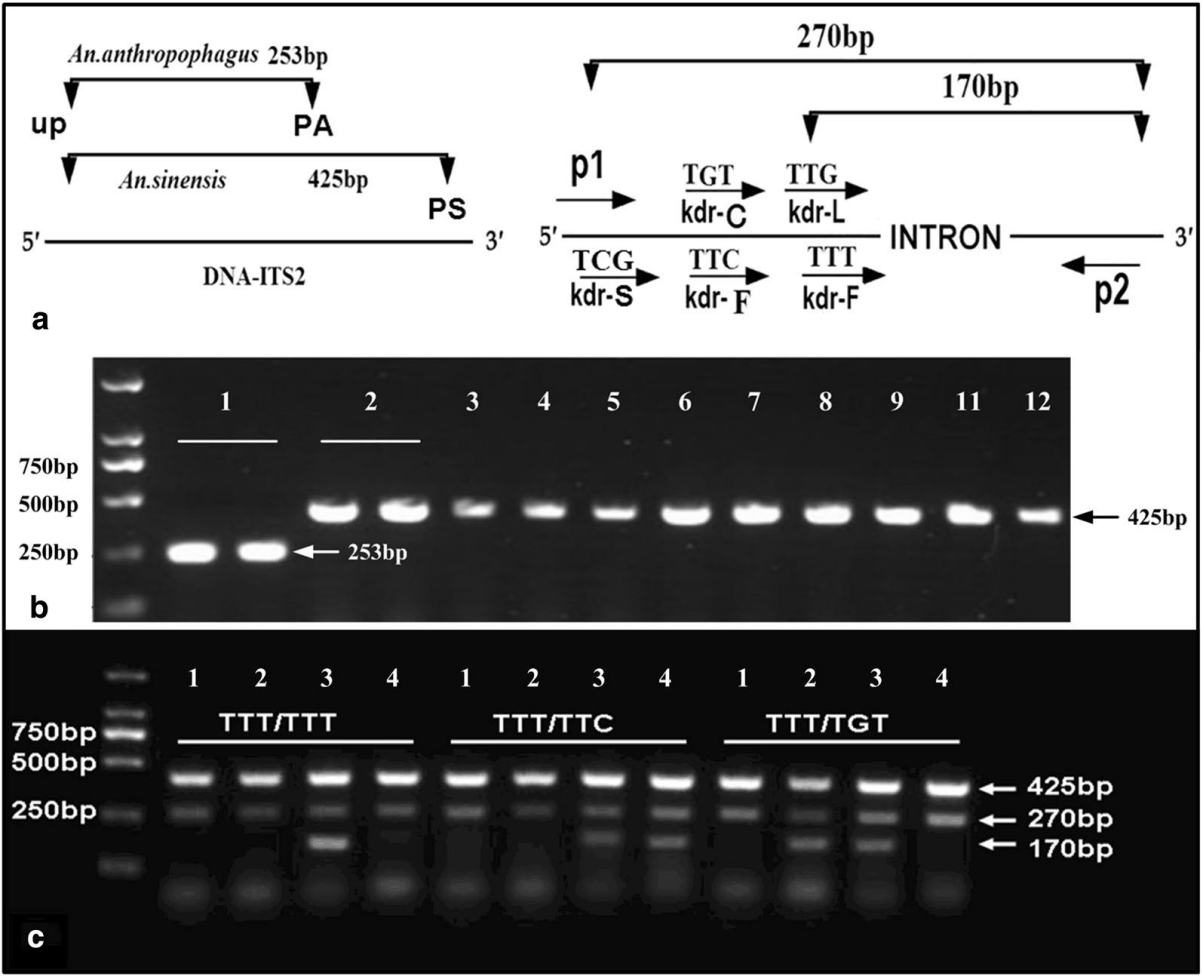
Schematic diagram of the the Multiple-PCR strategy was used to detect “TTG” to “TTT”, “TGT”, “TTC”and “TCG” mutations, predict the size of PCR products in the *para* sodium channel gene and identify the anopheles species. **a** P1–P2 with *kdr*-L, *kdr*-F, *kdr*-C and *kdr*-S indicate PCR primers. Paired-primer *kdr*-L and P2 amplifies a 170 bp fragment for the susceptible allele (for codon TTG). Primers pair *kdr*-F and P2 yields 170 bp fragments for the resistant TTT/TTC mutations (codon TTT and TTC). Similarly, primer pair *kdr*-S and P2 amplify a 170 bp fragment diagnostic of the TGT mutation (codon TGT). The primer pair P1 and P2 are allele-nonspecific outer primers. Paired primers of UP/PA and UP/PS were used to diagnostic the Anopheles specimen of *An. anthropophagus* and *An. sinensis*, respectively. **b** Species identification results (Lane 1: positive control of *An. anthropophagus*, Lane 2: positive control of *An. sinensis*, Lane 3–12: Partial sample amplification results). **c** Partial results by the Multiple-PCR showed the genotypes of TTT/TTT, TTT/TTC and TTT/TGT [Lane1: *kdr*-L(TTG), Lane2: *kdr*-C(TGT), Lane3: *kdr*-F(TTT), Lane4: *kdr*-F(TTC)]

Based on the work of Tan et al. [19] and Zhong et al. [25], with some additional modifications, a Multiple-PCR assay was developed to detect *kdr* genotypes and identify the *Anopheles* species (Fig. 2a). Two *Anopheles* species, *An. sinensis* and *Anopheles anthropophagus*, were sympatric in eastern and southern China, including Jiangsu and Anhui provinces. Because it is difficult to distinguish these species with anatomical keys, the PCR method on the basis of species-specific sequence differences in the ribosomal DNA internal transcribed spacer II was used to identify these species [26]. The diagnostic lengths of the species-specific fragments were 425-bp in *An. Sinensis*, based on the primers UP (5′-CCATGACGTACACAACTTG-3′) and PS (5′-GTTGTCCAGCCCGCTAACAT-3′), and 253-bp in *An. anthropophagus* based on the primers UP and PA (5′-GCTCCA TCTACACACA GCGT-3′) in the Multiple-PCR. *An. anthropophagus* has hardly been found in this area in recent years. Once the results of identification are suspected, we will pay attention to the sequencing results.

These five PCR reactions were almost identical except for different sense-specific primers. The first had a sense-specific primer (*kdr*-L: *5′*-TCCCGGTGGTAATTGGAAACTTG-3′) ending with the bases “TTG” at the 3′ end to detect the amplicon containing the codon “TTG”. The other four sense-specific primers ending with bases of “TGT” (*kdr*-C: 5′-ATGCGGTGGTAATTGGAAACTGT-3′), “TTT” (*kdr*-F: 5′-ATGCGGTGGTAATTGGAAACTTT-3′), “TCG” (*kdr*-S: 5′-ATGCGGTGGTAATTGGAAACTCG-3′) and “TTC” (*kdr*-F: 5′-TATGCGGTGGTAATTGGAAACTTC-3′) at the 3′end to detect the amplicons containing the special codons. Two additional non-specific outer primers, P1 (5′-ACCGTTGTCAAGTGCTGACCTGTCG-3′) and P2 (5′-TTGTTGTGTCATGCATCCCATTTCTTC-3′), were designed based on the sequences immediately downstream and upstream of the mutation sites.

Multiple-PCR diagnostic tests was performed in accordance with standard procedure with a total volume of 50 μl, consisting of 10× Buffer 5 µl, 80–100 ng genomic DNA 4 μl, 25 mM MgCl_2_ 2 μl, 10 mM each dNTP 2 μl, 1 μl KOD Plus polymerase, 4 μl primers and 32 μl ddH_2_O. For each template from individual strains, there were five PCR reactions. The first amplification included the UP, PA, PS and P1, *kdr*-L and P2 primers, the other four included the P1, P2 and *kdr*-F (TTT), *kdr*-F (TTC), *kdr*-C (TGT) or *kdr*-S (TCG). PCR conditions were one cycle of 93 °C for 4 min, then 35 cycles of 94 °C for 1 min, 55 °C for 30 s and 68 °C for 1 min, followed by one cycle of 68 °C for 7 min. PCR products were checked by electrophoresis on a 1.2% agarose gel in TAE buffer. The resulting bands were visualized following ethidium bromide staining. The size of the diagnostic PCR products for *kdr* alleles was 170 bp and the size of products for the nonspecific outer primers was 270 bp.

### Genotyping the *kdr* allele with the TaqMan assays

**Fig. 3:**
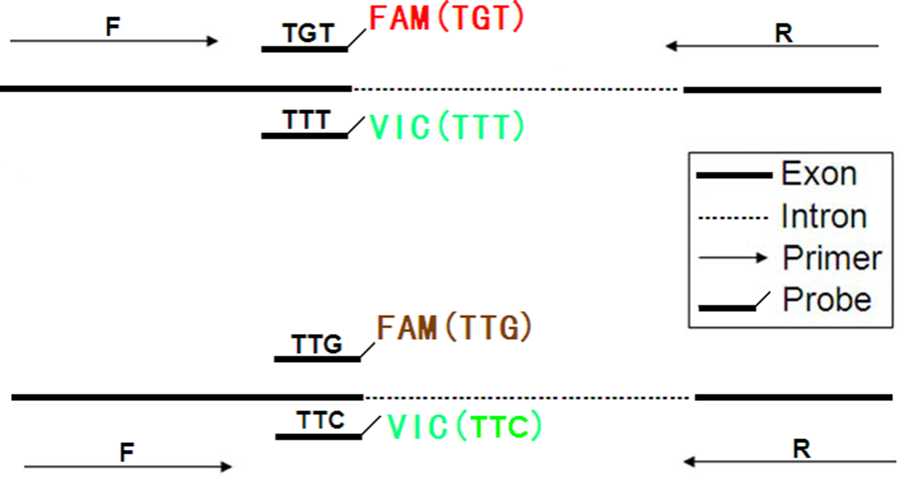
Schematic diagram of the TaqMan assay. Two parallel tests were used to genotype each DNA templates. The two tests were done in the same conditions except that one contained probe 1 and probe 3 with an outcome of red or green colour to detect the *kdr* allele of “TGT” and “TTT”, whereas the other contained probe 2 and probe 4 with an outcome of brown or green colour to detect the *kdr* allele of “TTG” and “TTC”

The TaqMan assays were designed to distinguish *kdr* allele types. The assay conditions used in this study were modified slightly from Chen et al. [17] but were not given in their original manuscript. Four probes and two non-specific out primers (D1 and D2) were designed with Primer Express™ Software Version 2.0 based on the *kdr* mutations of TTG (F-TCACCACCAAGTTTP-MGB)→TTT(V-CACCACAAAGTTTCP-MGB), TGT (F-CACCACACAGTTTCP-MGB) and TTC(V-CACCACGAAGTTTCP-MGB). No TTG→TCG mutation was found based on the results of direct sequence and Multiple-PCR. The probe sequences were designed to be labelled with a FAM fluorescent reporter or a VIC fluorescent reporter at the 5′ end and a minor groove binding (MGB) at the 3′ end. The minor groove binder provides more accurate allelic discrimination by increasing the T_M_ between matched and mis-matched probes [17]. Green curves in the first or second tube implied the existence of the “TTT” and “TTC” allele, respectively. A red curve in the first tube implied the existence of the “TGT” allele whereas a brown curve in the second tube implied the existence of a “TTG” allele. Fluorescence-based PCR was performed in an ABI Step One Real-time machine. Two parallel reactions in two tubes were used to detect and genotype *kdr* allele, the first tube included probe 1 and probe 2 and the second included probe 3 and probe 4 (Fig. 3). After initiation of the PCR, the FAM1 and FAM2, VIC1 and VIC2 reporters fluoresced red, brown and green respectively.

The amplifying systems on the ABI Step One Real-time machine were as follows: two parallel PCR reactions (10 μl) containing 1 μl of genomic DNA, 5 μl of TaqMan Universal Master Mix, 900 nM of each primer and 200 nM of two probes (probe 1 and probe 3 or probe 2 and probe 3). Samples were run at temperature cycling conditions of 2 min at 50 °C and 10 min at 95 °C followed by 40 cycles of 95 °C for 10 s and 60 °C for 40 s with a final extension at 60 °C for 30 s. The increase in VIC and FAM fluorescence was monitored in real time by acquiring each cycle on the yellow (530 nm excitation and 555 nm emission) and green channel (470 nm excitation and 510 emission) of the Rotor-Gene, respectively.

### Ethics statement

Pre-permission (April 2009–November 2012) was granted for mosquito observation, collection and field studies in Jiangsu and Anhui provinces as part of the Infective Diseases Prevention and Cure Project (No: 2012ZX10004219). For mosquito collection in rice paddies, oral consent was obtained from field owners in each location. These locations were not protected land, and the field studies did not involve endangered or protected species.

## Results

### Sampling sites and KT_50_ to three pyrethroid insecticides

Wild *An. sinensis* mosquitoes were collected from eleven geographically separated sites in Jiangsu and Anhui provinces, Eastern China (Fig. 1, Table 1). Four collection sites, FC, ES, ML and PZ were in Anhui province. Three of the remaining sites, YL, XZ and XN were in northern Jiangsu province and the other four were in southern Jiangsu province. Only female *An. sinensis* mosquitoes were received for the following steps. 72 mosquitoes were received molecular identification at XN, DT, BN, FC, CZ, PZ, and 66 at YL, 48 at SZ, 36 at DY, 49 at CS, 68 at WH.

KT_50_ values to three pyrethroid insecticides (beta-cypermethrin, delta-methrin and permethrin) ranged from 8.95 min to 33.73 min, 11.22 min to 37.03 min, and 16.13 min to 47.62 min, respectively. Resistance ratios (R/S) of KT_50_ to three pyrethroid insecticides ranged from 2.44 to 9.20-fold, 3.31 to 12.22-fold and 3.23 to 9.54-fold, respectively. Due to a shortage of specimens, mosquitoes collected at DY were only tested against beta-cypermethrin (3.72 to Beta-cypermethrin). The highest R/S were found in specimens from DT (9.20 to Beta-cypermethrin, 12.22 to Delta-methrin) and WH (9.52 to permethrin) (Table 2). The KT_50_ and R/S values showed resistance to pyrethroid insecticides in these adult population.

**Table 2:** Resistance characteristics (KT50 and R/S) of adults of a susceptible laboratory strain (S.S.) of *An. sinensis* adults and *An. sinensis* adults collected at sites in Anhui and Jiangsu provinces, China (see Fig. 1 and Table 1 for site locations)

Specimen origin	Beta-cypermethrin		Delta-methrin		Permethrin	
	KT50 (min)	R/S	KT50 (min)	R/S	KT50 (min)	R/S
S.S.	3.67	1.00	3.03	1.00	5.00	1.00
YL	8.95	2.44	12.55	4.14	22.22	4.44
XN	12.43	3.39	11.22	3.70	16.13	3.23
SZ	13.97	3.81	10.03	3.31	20.42	4.08
DT	33.73	9.20	37.03	12.22	43.30	8.64
DY	13.65	3.72	/	/	/	/
CS	16.75	4.56	14.62	4.83	20.17	4.03
BN	20.30	5.53	22.20	7.33	30.38	6.08
ES	26.27	7.16	27.57	9.10	28.65	5.73
ML	23.65	6.44	16.20	5.35	33.53	6.71
WH	26.87	7.32	27.82	9.18	47.62	9.52
PZ	14.50	3.95	16.97	5.60	27.17	5.43
Average	19.20	5.23	19.60	6.48	29.00	5.79

### DNA sequencing and *kdr* genotyping

The total of 120 DNA samples from sample sites and control population were provided for PCR. All the genomic DNA samples were sequenced to target the putative *kdr* loci in the VGSC gene and identify candidate site(s) for *kdr* mutations. The wild-type *kdr* codon sequence spanning position 1014 was TTG in *An. sinensis*. Three types of *kdr* mutations were present as TTG to TTT, TTC and TGT. TTG and TTC mutations caused L1014F (Leucine to Phenylalanine). TGT mutation caused L1014C and a Leucine to Cysteine substitution. The mutation (TTG–TTC) was first reported in Anhui province. No mutation of TTG→TCG which caused L1014S substitution was found. A total of six genotypes were identified in the three populations. Two types of homozygote genotypes were detected: TTT/TTT, TGT/TGT with frequencies 60.00% and 4.00%, four types of heterozygote genotypes were detected: TTG/TTT, TTG/TGT, TTT/TGT, and TTT/TTC with frequencies of 2.00%, 6.00%, 20.00% and 8.00%, respectively (Table 3).

**Table 3:** Percent sensitivity and specificity of three methods of *kdr* allele genetyping

Genotype	Genotyping methods		
	Sequence (n = 100)	Multiple-PCR (n = 100)	TaqMan-MGB (n = 100)
TTG/TTG	0.00	0.00	0.00
TTT/TTT	60.00	68.00	64.00
TGT/TGT	4.00	2.00	4.00
TTG/TTT	2.00	2.00	2.00
TTT/TGT	20.00	22.00	18.00
TTG/TGT	6.00	2.00	8.00
TTT/TTC	8.00	4.00	4.00
Sensitivity (%)	/	94.00	98.00
Specificity (%)	/	94.00	100.00

### Species identification and genotyping results with multiple-PCR assay

In order to improve the work efficiency, a multiple-PCR amplification method was established to complete species identification and genotyping in one amplification reaction.

### Species identification

A total of 709 DNA samples were identified as *An. sinensis* by a species diagnostic PCR assay adapted from Ma et al. [27]. The lack of amplification of a 253-bp fragment indicated the absence of *An. anthropophagus*, therefore, all tested mosquitoes were deemed to be *An. sinensis* (Fig. 2b). This is consistent with the known geographic distribution of species within the *An. anthropophagus* complex in Jiangsu and Anhui provinces [19].

### *kdr* genotyping results

After optimization, the Multiple-PCR diagnostic test assay was capable of detecting homozygous (L/L) and heterozygous (L/C, L/F, C/F, F/F and C/C) genotypes of the L1014F and L1014C substitutions in the sodium channel gene. The product amplified by the two non-specific outer primers P1 and P2 was 270-bp. The product amplified by the four-specific paired-primers was 170-bp. Because of the absence of TTG–TCG mutation, four multiple-PCR reactions were performed to identify the genotypes (Fig. 2c). All the six genotypes were identified with multiple-PCR method and the mutation frequencies were examined (TTT/TTT 68.00%, TGT/TGT 2.00%, TTG/TTT 2.00%, TTG/TGT 2.00%, TTT/TGT 22.00%, TTT/TTC 4.00%) (Table 3). The results inferred from these diagnostic amplicons were a little lower in specificity (94%) and sensitivity (94%) than those obtained by direct sequencing (Table 3).

### Genotyping results with the TaqMan assay

**Fig. 4:**
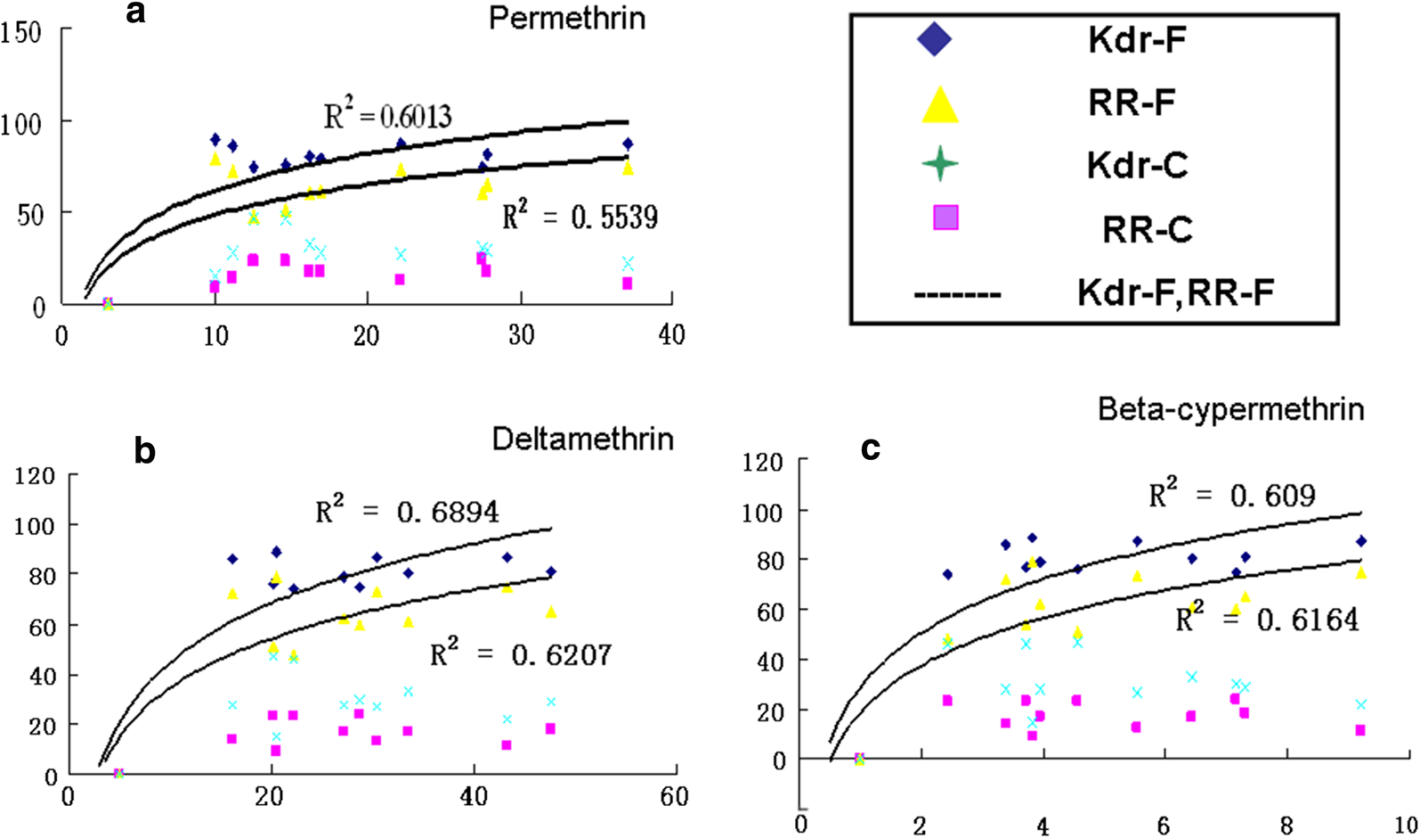
Correlation of the R/S values of betamethrim, deltamethrin and permethrim with *kdr* frequencies and genotypic frequencies.** a** Correlation between the different *kdr* allele requencies and R/S value of betamethrin on *An. sinensis* populations.** b** Correlation between the different *kdr* allele requencies and R/S value of deltamethrin on *An. sinensis* populations.** c** Correlation between the different *kdr* allele requencies and R/S value of permethrin on *An. sinensis* populations

After minimal optimization using templates of known genotype, both the *kdr*-F and *kdr*-C TaqMan assays demonstrated excellent discrimination of the two resistance alleles. Each template was examined with four probes in two parallel reactions and the outcome from a Step-one real-time PCR machine was displayed in different colours. Amplified curves of two parallel reactions were used to determine the genotype of each DNA sample. Because *An. sinensis* is a diploid organism, no more than two coloured curves can appear in the two parallel tubes. Six genotypes of the *kdr* allele were detected (Fig. 4). The specific probe for the wildtype allele labelled with FAM fluorescence and the second specific probe for the mutant allele *kdr*-F labelled with VIC fluorescence were indicative of the L1014F substitution (Fig. 4e). A substantial increase in FAM fluorescence for the mutant allele *kdr*-C and a substantial increase in VIC fluorescence for the mutant allele *kdr*-F indicated the heterozygote mutant allele 1014F/C (Fig. 4f). A substantial increase in FAM fluorescence for the wildtype allele L1014 and a substantial increase in FAM fluorescence for the mutant allele *kdr*-C indicated a heterozygote mutant allele L1014C (Fig. 4d). Individuals homozygous for the wildtype allele and the two *kdr* mutant alleles displayed an increase in either VIC or FAM fluorescence (Fig. 4a–c).

To help score genotypes, the Rotor–Gene software allows endpoint fluorescence values for the two dyes to be automatically corrected for background and plotted against each other in bi-directional scatter plots. The clustering of samples in scatter plots in addition to real-time fluorescence traces allowed easy and accurate genotype scoring. The results of genotyping the 50 samples on the reference plate showed that the real-time TaqMan assay was sensitive, with only two failed reactions. The mutation frequencies were calculated and showed in Table 3 (TTT/TTT 64.00%, TGT/TGT 4.00%, TTG/TTT 2.00%, TTG/TGT 8.00%, TTT/TGT 18.00%, TTT/TTC 4.00%). The genotyping outcomes obtained by the TaqMan assay were as specific as those obtained by direct sequencing (100%), but a little less sensitive (98%) (Table 3).

### Distribution of *kdr* allele frequencies in natural populations

Direct sequencing was used to validate the results obtained with the Multiple-PCR and TaqMan assays. Both methods can effectively detected L1014-F/C substitutions and the sensitivity and specificity were found to exceed 94% in all cases (Table 3). But the Multiple-PCR assay is much easier to operate and with less costs, so it is recommended as a common genotyping assay for *kdr* monitoring. Finally, a total of 709 samples from specimens collected at the eleven sites were tested for the *kdr* mutant allele with multiple-PCR (Fig. 1, Table 4).

**Table 4:** Allelic and genotype *kdr* frequencies of adult *An. sinensis* from a susceptible laboratory strain (SS) and those of adult *An. sinensis* specimens collected at sites in Anhui and Jiangsu provinces, China (see Fig. 1 and Table 1 for site locations)

Specimen origin	SS	YL	XN	SZ	DT	DY	CS	BN	PZ	ES	ML	WH
Sample size (n)	50	66	72	48	72	36	49	72	72	72	72	68
Frequency of *kdr* allele (%)												
TTG (L1014)	100	33	0	2	2	0	1	0	1	3	1	4
TTT/TTC (1014F)	0	74	86	89	87	77	76	87	75	80	81	79
TGT (1014C)	0	23	14	9	11	23	23	13	24	17	18	17
Frequency of *kdr* genotype (%)												
L/L (TTG/TTG)	100	0	0	0	0	0	0	0	0	0	0	0
L/F (TTG/TTT)	0	6	0	4	2	0	2	0	0	6	3	6
L/C (TTG/TGT)	0	0	0	0	1	0	0	0	3	0	0	3
F/F (TTT/TTT) F/F (TTT/TTC)	0	48	72	79	75	54	51	73	60	61	65	62
C/C (TGT/TGT)	0	0	0	2	0	0	0	0	7	0	3	1
F/C (TTT/TGT)	0	46	28	15	22	46	47	27	30	33	29	28

Based on the presence or absence of *kdr* alleles, individual mosquitoes were classified as homozygous susceptible (SS), homozygous resistant (RR), or heterozygous (RS). No homozygous susceptible (SS) specimens were detected. Neither of the two resistant alleles was present in any of the control specimens. PCR assays revealed clear differences in overall *kdr* allelic frequency between samples from wild and control specimens. All control specimens were homozygous susceptible but *kdr* frequencies in wild caught specimens ranged from 9 to 86%. The *kdr* alleles found were mainly of the *kdr*-F/F and *kdr*-F/C genotypes. Only a small portion (1–7%) of specimens possessed *kdr*-C homozygous (RR-C/C) *kdr* alleles, with 1–6% of *kdr*-C and *kdr*-F specimens collected from five sample sites being of the heterozygous (RS-C and RS-F) *kdr* genotype. *Kdr*-*F* and *kdr*-C frequencies ranged from 74 to 89% and 9 to 24%, respectively. The frequency of RR-F/F and RR-F/C ranged from 48 to 79% and 15 to 47%, respectively (Table 4).

### Substitution frequencies in response to beta-cypermethrin, deltamethrin and permethrin

Regression analysis revealed a significant correlation between KT_50_ estimates of susceptibility to beta-cypermethrin and the frequency of *kdr*-F (R^2^ = 0.615), RR-F/F (R^2^ = 0.614), *kdr*-(F + C) (R^2^ = 0.62). There was also a correlation between deltamethrin and permethrin resistance and mutative allele frequencies; *kdr*-F (R^2^ = 0.698), RR-F/F (R^2^ = 0.624), *kdr*-(F + C) (R^2^ = 0.47), *kdr*-F (R^2^ = 0.608), RR-F/F (R^2^ = 0.554), *kdr*-(F + C) (R^2^ = 0.47), respectively. No significant correlations were found between the other *kdr* genotype frequencies and R/S values (Table 5).

**Table 5:** Correlation between KT50 and frequencies of *para*-*kdr* gene and genotype

Insecticides	Genotype	Equation	R^2^
Beta-cypermethrim	*kdr*-F	Y = 31.593Ln(x) + 28.159	0.615
	RR-F/F	Y = 27.42Ln(x) + 18.506	0.614
	*krd*-(F + C)	Y = 37.527Ln(x) + 34.491	0.62
Deltamethrin	*kdr*-F	Y = 21.585Ln(x) + 37.984	0.698
	RR-F/F	Y = 16.767Ln(x) + 30.3498	0.624
	*kdr*-(F + C)	Y = 25.758Ln(x) + 46.78	0.47
Permethrim	*kdr*-F	Y = 21.585Ln(x) + 37.984	0.608
	RR-F/F	Y = 16.767Ln(x) + 30.349	0.554
	*kdr*-(F + C)	Y = 25.758Ln(x) + 46.78	0.47

## Discussion

In recent years, the main control strategy for *An. sinensis* is long-lasting spraying pyrethroid insecticides indoors and using of impregnated mosquito nets, for their habitat of entering human dwellings in the late afternoon and some of them staying inside, [28]. The increasing use of pyrethroid insecticides has led to the increasing prevalence of insecticide resistance in *An. sinensis* populations [29]. Pyrethroid resistance in *Anopheles* populations has the potential of seriously compromise malaria control efforts. A recent report examining the effectiveness of using ITNs at two sites in Benin has provided clear evidence that pyrethroids insecticides failed to control an *An. gambiae* population with high *kdr* levels [30]. Previous studies have found that the frequency of *kdr* mutations were associated with the actual resistance level to pyrethroid insecticides. So, it is necessary to have a resistance monitoring before spraying insecticides. However, a convenient genotyping assay is urgent to monitor the *kdr* alleles.

Nowdays, larval bioassays ware generally used for monitoring insecticide resistance in Chinese satellite CDC (Centers for Disease Control and Prevention) branches. Insecticide resistance is different between larval and adult life stages and that resistance character is not always transferred to the adult stage and vice versa. Different life stages always show difference in resistance level. Larvae live in the water of paddy fields and consequently absorb insecticides through the mouth, skin and stigma. There was an evidence that metabolic enzymes play an important role in the resistance of *Culex piens pallens* larvae to pyrethroid insecticides [31]. The resistance of adult mosquitoes from Jiangsu province to beta-cypermethrin was far below than that of larvae. In China, the main strategies of malaria prevention are IRS and ITNs with pyrethroid insecticides used on adult *An. sinensis* population. Therefore, instruction of spraying insecticides should be made based on the resistance phenotype of adult *An. sinensis* population. This research aim to reveal the association between resistance phenotype and *kdr* mutation and help to change the traditional practice.

In this study, 11 field population of *An. sinensis* were collected from Jiangsu and Anhui province from 2009 to 2012. Using the Wire-Ball bioassay, the half knockdown time (KT_50_) of all adult *An. sinensis* samples were tested and the resistance index of specimens from the eleven sampling sites ranged from 2.4 to 12.2, with average resistance to beta-cypermethrim, deltamethrin and permethrim being 5.23, 6.48 and 5.79, respectively. The 21 of 31 R/S values from the eleven sampling sites were over 4.0, indicating that mosquitoes at these sites meet, or exceed, the WHO threshold for pesticide resistance (GB/T., 2010). These results suggest that the *An. sinensis* populations in those areas involve high pyrethroid resistance. The past controlling method with pyrethroid insecticides might be fail.

Knockdown resistance (*kdr*) is known to confer cross-resistance to DDT and pyrethroids [14]. The Leu/Phe substitution and prior reports of the *kdr* mutation have been implicated in the development of pyrethroid resistance in several mosquito species, including *An. gambiae* [32], *Culex pipiens pallens* [33] and *Culex pipiens quinquefasciatus* [34, 35]. Previous researches have found high frequencies of 1014F/C and 1014F/S substitutions in *An. sinensis* populations in Jiangsu and Guangxi provinces [19, 22]. The sequencing results showed that the L1014F substitution was the main *kdr* genotype with a frequency ranging from 74 to 89% in the test areas. The *An. sinensis* samples collected from all eleven collection sites showed significant resistance to all three pyrethroid, suggested that there was a relationship between the L1014 substitution and pyrethroid resistance phenotype. The L1014F substitution showed a strong positive correlation with KT_50_ (R^2^ = 0.55–0.69) and a significantly high frequency (74% to 89%) corresponding to the level of resistance level to pyrethroids in specimens from all collection sites (2.4- to 12.2-fold). This finding therefore supports previous studies and suggested that the L1014F substitution was the key mutation and responsible for pyrethroid resistance. The alternative leucine to cysteine (L1014C) substitution at the same location occurred at relatively low frequency (24% to 9%), which, together with its much lower correlation with KT_50_, suggests that it doesn’t play an important role in pyrethroid resistance. The high L1014F substitution frequency in adults brings higher beta-cypermethrin resistance than that in the larvaes in the XZ population. Beta-cypermethrin resistance was more significantly correlated with *kdr* frequencies than the other two pyrethroids. These suggest that beta-cypermethrin played a more important role in pyrethroid resistance in *An. sinensi*s. Based on the positive correlation between KT_50_ and *kdr*-F and *kdr*-(F + C) frequencies, with the results of previous studies on other mosquito species, we consider that *kdr* mutation screening is an excellent indicator of pyrethroid resistance in adult *An. sinensis.* Due to the large population size and wide species range in China, the *kdr* gene in *An. sinensis* populations (the dominant *Anopheles* mosquito in China) shows highly polymorphic [21]. The different allele statuses of *kdr* genes and resistance mechanisms in the *An. sinensis* suggest the importance of monitoring insecticide susceptibilities and the resistance of target vectors in control programmes. Effective resistance monitoring that is based on species-specific insecticide bioassay tests, molecular studies of allele diversity, origins of insecticide resistance, and minor resistance mechanisms (behavioural and cuticular resistance) will be crucial for building successful malaria vector control programmes that can explain and predict the development and spread of insecticide resistance traits [21].

The results suggest that the *kdr* allele increases in frequency mainly as a result of transfer between individuals in the same population. The using of different insecticides plays an important role in determining the frequency of the *kdr* allele in *An. sinensis* populations. It is well known that there was a high correlation between the resistant phenotypes and *kdr* frequencies, allegro monitoring of *kdr* allele mutations is the key step in the malaria control. It is important to be able to monitor the spread of the alleles that confer resistance to different classes of insecticides and use this information to determine which insecticides are most appropriate for a given vector. Here, the different methods were tested to identify the *kdr* mutation frequency. This not only allows the calibration of methods used by different CDC branches in China, but also comparison of the sensitivity of different methods of detecting *kdr* mutations. The TaqMan-PCR assay is a PCR method employing oligonucleotide probes that are dual-labelled with a fluorescent reporter dye and a quencher molecule. Amplification of the probe-specific product causes cleavage of the probe, generating an increase in reporter fluorescence as the reporter dye is released from the quencher. By using different reporter dyes, cleavage of allele-specific probes can be detected in a single PCR [36]. Although it requires relatively expensive instruments, this assay gives precise results and is recommended for use in provincial CDC and research institutes in China. The Multiple-PCR assay is an excellent method of resistance detection that is ideal for grassroots CDC branches due to its simplicity and low cost. Genotyping the *kdr* allele and identitying the anopheles species in the same amplifying reaction gave the higher efficiency in resistance monitoring. It is difficult to debugging the amplifying reaction of multiple-PCR because a slight increase or decrease of a single primer would lead to big change in the results. So, multiple methods should be combined for the detection of *kdr* mutations.

## Conclusions

In this study, the distribution of insecticides resistance for adult *An. sinensis* to three pyrethroid insecticides in Jiangsu and Anhui provinces has been mapped, Eastern China. This result suggests that the pyrethroid resistance of *An. sinensis* populations in those areas was serious and more attention should be paid to it. The frequency of target site (L1014) mutations conferring pyrethroid resistance had been investigated with multiple methods and the multiple-PCR assay was chosen to be the common genotyping assay for *kdr* monitoring. Further in-depth experiments are required to investigate the current epidemiological and evolutionary dynamics of malaria vectors, especially *An. sinensis* in the eastern China. Meanwhile, these efforts will be one of the prerequisite steps needed to establish effective long-term vector control strategies in the eastern China.

## Abbreviations

CDC: Centers for Disease Control and Prevention
ddH_2_O: double distilled water
DDT: dichloro-diphenyl-trichloroethane
IRS: indoor residual spraying
ITNs: Insecticide-treated mosquito nets
*kdr*: knockdown resistance
KT_50_: half knockdown time
MGB: minor groove binding
PCR: polymerase chain reaction
R/S: resistance ratios
RR: homozygous resistant
RS: heterozygous
SS: homozygous susceptible
VGSC: voltage-gated sodium channel
WHO: World Health Organization
