# Correction to: The polymorphism and geographical distribution of knockdown resistance of adult *Anopheles sinensis* populations in eastern China

**DOI:** 10.1186/s12936-019-2815-x

**Published:** 2019-05-28

**Authors:** Wei-Long Tan, Chun-Xiao Li, Rui-Chen Lv, Yan-De Dong, Xiao-Xia Guo, Dan Xing, Ming-hao Zhou, Yan Xu, Hong-liang Chu, Gang Wang, Chang-qiang Zhu, Jun Sun, Tong-Yan Zhao

**Affiliations:** 1 State Key Laboratory of Pathogen and Biosecurity, Beijing Institute of Microbiology and Epidemiology, Beijing, 100071 China; 2 Department of Vector Control, Huadong Research Institute for Medicine and Biotechnics, Nanjing, 210002 Jiangsu China; 3 Department of Vector Control, Jiangsu Center for Disease Prevention and Control, Nanjing, Jiangsu China

## Correction to: Malar J (2019) 18:164 10.1186/s12936-019-2793-z

Please be advised that since publication of the original article [1] the authors have flagged that they omitted to provide the up-to-date version of Fig. 1 and, as such, the wrong version of Fig. 1 is present in the article.

Please see below for the correct (up-to-date) version of the figure:

**Fig. 1:**
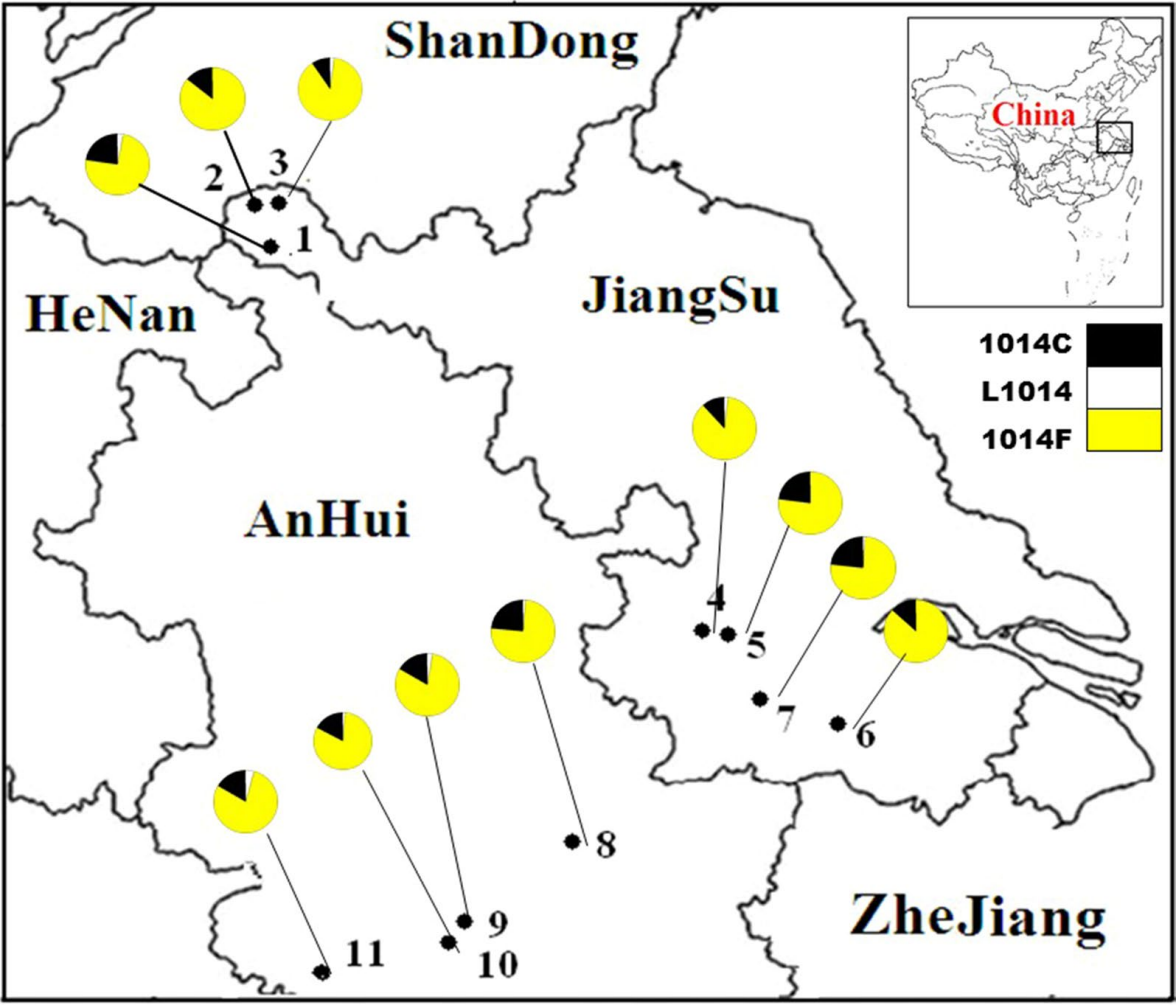
The location of collection sites of adult *Anopheles sinensis* specimens and distribution of *kdr* allele. 1: YL (Yunlong); 2: XN (Xiaonian); 3: SZ (Shazhuang); 4: DT (Dangtu); 5: DY (Danyang); 6: BN (Benniu); 7: CS (Changshu); 8: FC (Fanchang); 9: CZ (Chizhou); 10: WH (Wanghe); 11: PZ (Panzheng). In the colour circles, different colour indicated different substitutions in percentage

The authors apologize for this error.
